# Blue Sulphur Springs, Virginia

**Published:** 1835

**Authors:** 


### IX.—Blue Sulphur Spring.

Dr. Patrick, of Charleston, Va., has favored us with some bottles of water from a mineral spring, hitherto not much frequented. It is situated about twenty miles west of the White Sulphur, and the same distance north-west from the Red Sulphur Spring, Greenbriar county, Virginia.

We put a quantity of this water into the hands of Mr. Riddell, advantageously known to the readers of our journal as an able chemist, mineralogist, and botanist, who being at the time about to set off on a tour of observation, could not subject it to a full and accurate analysis. He obtained, however, the following results; and has promised, on a future day to make a more thorough examination.

The following are the ingredients:

SULPHURETTED HYDROGEN.

SULPHATE OF MAGNESIA, or *Epsom Salt.*

SULPHATE OF SODA, or *Glauber Salt*, a trace.

SULPHATE OF LIME.

MURIATE OF SODA.

MURIATE OF LIME.

BICARBONATE OF MAGNESIA.

BICARBONATE OF LIME ?

A spring holding such ingredients, in solution, cannot fail to prove highly beneficial in chronic diseases of the liver, stomach and bowels.

Western Virginia abounds in mineral springs, exceedingly diversified in their qualities. It is, indeed, unequalled in the United States, and not surpassed perhaps by any district of country in the world.

